# Warming Arctic summers unlikely to increase productivity of shorebirds through renesting

**DOI:** 10.1038/s41598-021-94788-z

**Published:** 2021-07-27

**Authors:** Sarah T. Saalfeld, Brooke L. Hill, Christine M. Hunter, Charles J. Frost, Richard B. Lanctot

**Affiliations:** 1 U.S. Fish and Wildlife Service, Migratory Bird Management, 1011 East Tudor Road, MS 201, Anchorage, AK 99503 USA; 2 Department of Biology and Wildlife, University of Alaska Fairbanks, P.O. Box 756100, Fairbanks, AK 99775 USA; 3 Present Address: Coastal Bend Bays and Estuaries Program, 615 N. Upper Broadway, Suite 1200, Corpus Christi, TX 78401 USA; 4 Present Address: Department of Conservation, Biodiversity Group, Private Bag 5, Nelson, 7010 New Zealand

**Keywords:** Climate-change ecology, Conservation biology

## Abstract

Climate change in the Arctic is leading to earlier summers, creating a phenological mismatch between the hatching of insectivorous birds and the availability of their invertebrate prey. While phenological mismatch would presumably lower the survival of chicks, climate change is also leading to longer, warmer summers that may increase the annual productivity of birds by allowing adults to lay nests over a longer period of time, replace more nests that fail, and provide physiological relief to chicks (i.e., warmer temperatures that reduce thermoregulatory costs). However, there is little information on how these competing ecological processes will ultimately impact the demography of bird populations. In 2008 and 2009, we investigated the survival of chicks from initial and experimentally-induced replacement nests of *arcticola* Dunlin (*Calidris alpina*) breeding near Utqiaġvik, Alaska. We monitored survival of 66 broods from 41 initial and 25 replacement nests. Based on the average hatch date of each group, chick survival (up to age 15 days) from replacement nests (Ŝ_*i*_ = 0.10; 95% CI = 0.02–0.22) was substantially lower than initial nests (Ŝ_*i*_ = 0.67; 95% CI = 0.48–0.81). Daily survival rates were greater for older chicks, chicks from earlier-laid clutches, and during periods of greater invertebrate availability. As temperature was less important to daily survival rates of shorebird chicks than invertebrate availability, our results indicate that any physiological relief experienced by chicks will likely be overshadowed by the need for adequate food. Furthermore, the processes creating a phenological mismatch between hatching of shorebird young and invertebrate emergence ensures that warmer, longer breeding seasons will not translate into abundant food throughout the longer summers. Thus, despite having a greater opportunity to nest later (and potentially replace nests), young from these late-hatching broods will likely not have sufficient food to survive. Collectively, these results indicate that warmer, longer summers in the Arctic are unlikely to increase annual recruitment rates, and thus unable to compensate for low adult survival, which is typically limited by factors away from the Arctic-breeding grounds.

## Introduction

The Arctic has long been recognized as an important breeding location for avian species, with ~ 200 species of shorebirds, landbirds, ducks, geese, raptors, and grouse present^1^. Shorebirds are the dominant taxa across all regions of the Arctic; many of which are experiencing population declines^1^. The effects of climate change in the Arctic, including earlier, warmer, and longer summers^2–4^, will impact avian species and their habitats in a variety of ways^1,5^. For insectivorous species, such as shorebirds, one of the main hypothesized effects of earlier summers is a phenological mismatch between the timing of chick hatch and the availability of their invertebrate prey^6–10^. While several studies have shown that both Subarctic- and Arctic-breeding shorebirds^11–14^ and their invertebrate prey^15,16^ have advanced their phenologies in recent decades, most studies have shown that advancements in shorebird hatch dates have not kept pace with earlier availability of invertebrate prey^11,14^. This disparity is likely because shorebirds time their northward, long-distance migrations using a combination of relatively constant endogenous and photoperiod cues^17,18^, whereas the availability of their invertebrate prey is dictated by local climate conditions^15,19,20^. While it is generally assumed that greater phenological mismatch will result in reduced survival of shorebird chicks^6,7,10^ (but see McKinnon et al.^21^), few studies have investigated survival of chicks related to climatic and food conditions within the Arctic^10,22,23^. This is most likely due to logistical constraints in sampling chicks that leave nest sites within hours of hatching^24^, are highly mobile^25^, are difficult to relocate^26^, and often hide or remain motionless in the presence of a predator (or researcher)^27^. Instead, most studies have relied on growth rates of chicks when investigating the impacts of phenological mismatch^6,7,9,21,28,29^. However, the relationship between growth and survival of chicks is not necessarily straightforward; the assumption that slower growth leads to lower survival may not be true if undernourished chicks can simply grow more slowly over a longer period without compromising survival. Other factors, such as predation rates, may also have a greater effect on survival of chicks independent of growth rates. These uncertainties make studies of chick survival, rather than chick growth, important for assessing impacts of a changing climate.

A complicating factor in assessing climate change effects on annual productivity is how the predicted warmer, longer summers will affect the ability of adults to double brood (i.e., initiation of a second clutch of eggs after successfully raising young from the first clutch) or replace a nest if the first one fails, potentially producing young from a subsequent nest or several nests. While double brooding is not thought to occur in shorebirds breeding in the Arctic and only rarely in the Subarctic (see e.g., Jamieson^26^), ample evidence exists that many species renest (i.e., replace nests that are lost within a breeding season; see Naves et al.^30^ and references therein). While the extent to which individuals replace lost nests appears dependent on the time available to nest, other factors including parental condition, environmental conditions, and mating strategy may be also important (see Naves et al.^30^ and Swift et al.^31^ for a review of this topic). In one of the few experimental studies to address the potential to renest, Gates et al.^32^ found that on average, 75% (SE = 6%) of female Dunlin (*Calidris alpina*) renested when their first clutch was removed. While successful renesting may increase an individual’s annual reproductive success, adults that lay a second clutch of eggs may suffer from increased energetic costs, greater predation risks, less time to prepare for southward migration, and ultimately lower lifetime reproductive success^31^. Additionally, the reproductive value (e.g., number of hatchlings or fledglings) from replacement nests may not be equal to that of initial nests, as replacement nests in temperate and subarctic regions often have both lower nest and chick survival as compared to initial nests^10,26,31^. However, additional data are needed from the Arctic, where climate change may be increasing the ability of adults to renest^33^. Warmer and longer summers may also offer physiological relief to chicks (i.e., warmer temperatures that reduce thermoregulatory costs), which could enhance the likelihood of them surviving from both initial and replacement nests^21^. Given these potential outcomes, an assessment of the reproductive value of replacement nests is vital to our understanding of the implications of climate change on the annual productivity of shorebirds.

Understanding what factors influence survival of chicks is also important for developing accurate demographic models needed to estimate trends in shorebird populations. As shorebirds tend to be long-lived, with rather invariable clutch size (i.e., generally 4 eggs), adult survival rates, which are typically limited by factors away from the Arctic-breeding grounds, have larger effects on population growth than similar changes in survival of juveniles or chicks^34–37^. However, studies have also found both juvenile^34,37^ and chick survival^36^ to be important drivers (albeit to a lesser degree than adult survival) in shorebird population models. Sparse data on juvenile and chick survival rates has necessitated the use of generalized estimates when developing shorebird population models; weakening the validity of the models and the trend estimates they produce^34^. Therefore, species- and population-specific estimates of chick survival, especially from studies that take into account potential effects of climate change, are needed to ensure the accuracy of demographic models and resulting trends in population growth. Indeed, climate-driven increases in chick survival might be important for compensating for low adult survival experienced on the nonbreeding grounds. This may be especially important for many shorebird species, such as Dunlin, in which population declines have been linked to low annual adult survival^38^ as a result of non-breeding habitat loss^39–42^.

**Figure 1:**
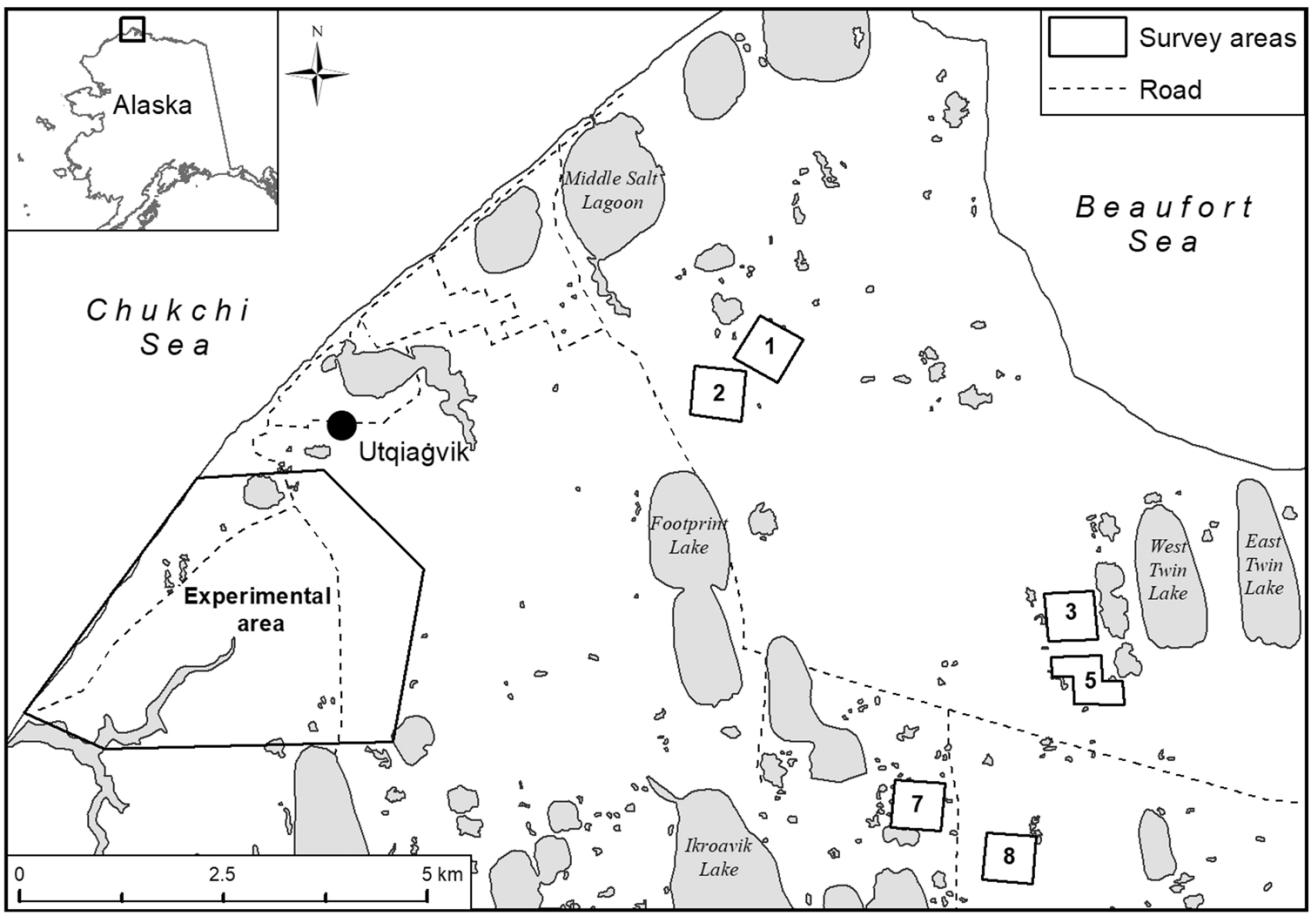
Location of unmanipulated (i.e., the six 36-ha numbered plots) and experimental study areas used to investigate Dunlin chick survival near Utqiaġvik, Alaska, 2008–2009. (Generated by ArcGIS 10.8.1, URL: http://www.esri.com/software/arcgis/arcgis-for-desktop).

In this study, we investigated survival of chicks from initial and replacement nests of the *arcticola* subspecies of Dunlin breeding on Alaska’s North Slope (Fig. 1). We capitalized on an existing study wherein a portion of initial nests were experimentally removed to determine rates of renesting^32^. We used marked and radio-tagged parents and young from both undisturbed initial nests and experimentally-induced replacement nests to monitor chick survival. Specifically, we estimated daily survival of chicks and determined whether survival was related to invertebrate abundance, temperature, year, chick age, and hatch date (and by association initial and replacement nests).

We predicted survival of shorebird chicks would (1) increase with invertebrate abundance, as chick growth and survival often decrease with lower food availability^6,7,10,21,28,43–45^; (2) decrease with lower temperatures due to lower invertebrate activity (i.e., less food for chicks^15,20,44,46^) and greater thermoregulatory costs, especially in younger chicks^10,21,23,44,47^; (3) vary between years as a result of annual variation, (4) increase with chick age, as previous studies have documented lower survival rates in younger chicks, likely a result of susceptibility to predation and extreme weather events^22,48^, and (5) decrease later in the season (and by association be lower in replacement nests), as chicks hatching earlier in the season often have greater growth and survival rates than those hatching later ^6,10,22,47–49^. This may be due to synchrony with food availability, although seasonal variation in other factors such as predation, weather, or parental care may also impact seasonal survival rates^9,10,22^.

## Results

### Chick monitoring

We removed 89 clutches (60 during early incubation and 29 during late incubation), from which, we found 63 replacement nests (48 from the early treatment and 15 from the late treatment). In total, we monitored 66 broods: 41 (19 in 2008, 22 in 2009) from initial nests, 18 (13 in 2008, 5 in 2009) from early replacement nests, and 7 (6 in 2008, 1 in 2009) from late replacement nests. Chicks hatched from 27 June to 24 July (2008: 1–24 July, 2009: 27 June–21 July), with chicks from initial nests tending to hatch earlier (27 June–15 July, mean = 3 July, SE = 0.7 days) than chicks from early and late replacement nests (early nests: 10–17 July, mean = 13 July, SE = 0.6 days; late nests: 21–24 July, mean = 22 July, SE = 0.5 days). As we present results using the average hatch date of replacement nests (i.e., 15 July; see below), it should be noted, that this value was similar to the average hatch date of natural replacement nests found from 2003 to 2019 (i.e., 18 July). We successfully radio tagged two chicks from all broods. However, one chick from a single brood failed to leave the nest bowl and its death was attributed to handling and marking; therefore, it was excluded from all further analyses. We monitored chicks from hatching to failure or fledging for a total of 30 days in 2008 (1–30 July) and 41 days in 2009 (27 June–6 August).

**Figure 2:**
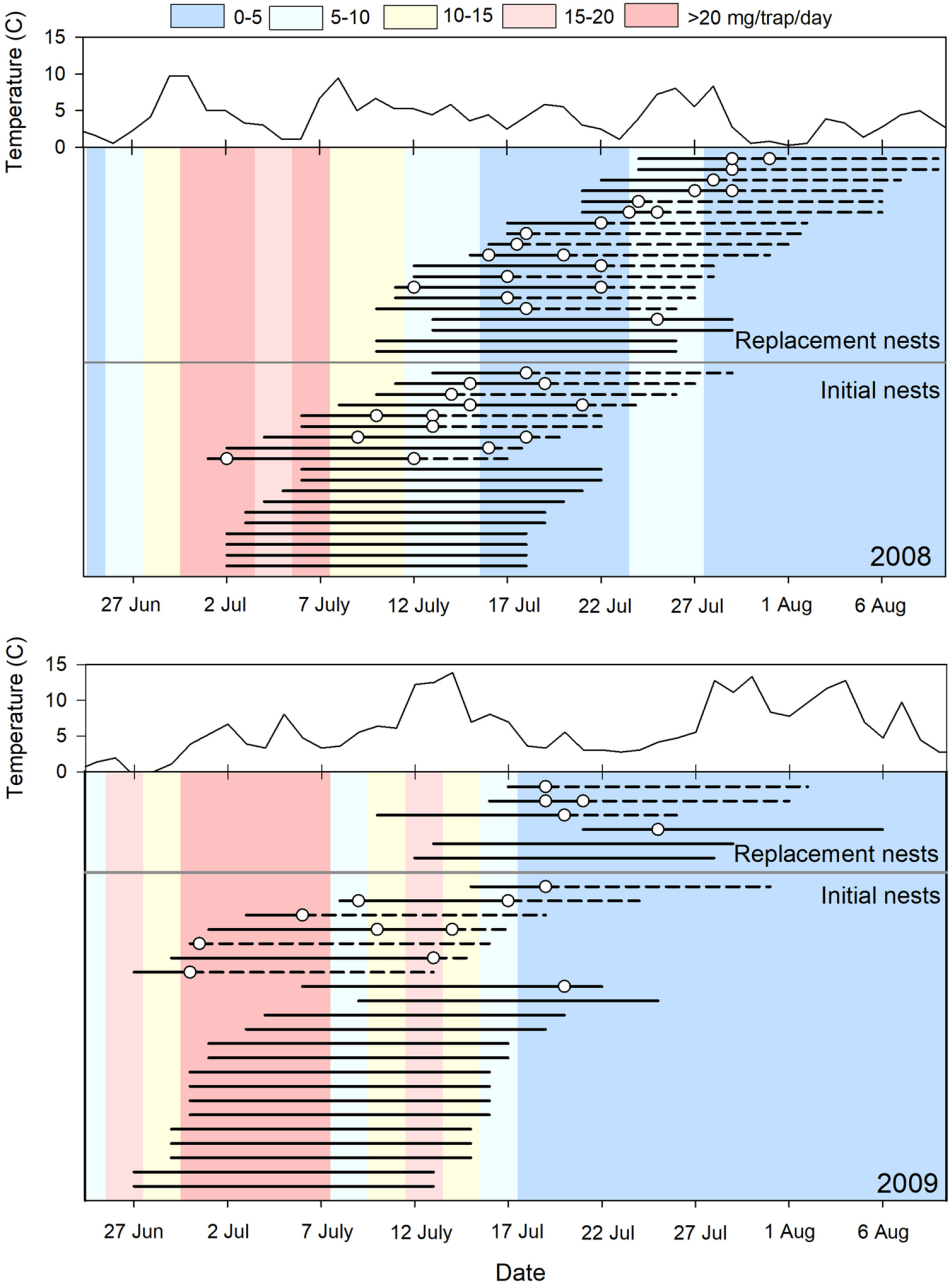
Phenology of Dunlin broods from initial (below solid gray horizontal line) and replacement nests (above solid gray horizontal line) in relation to invertebrate availability (background colors) and temperature (line graph) near Utqiaġvik, Alaska, 2008–2009. Each line represents phenology of a given brood from hatching to fledging (16-day period) with broods ordered by fate (i.e., broods with mortality of both chicks are at the top) and hatch date. White dots indicate dates when radio-tagged chicks died (i.e., median date between the last date seen alive and date found dead). Dashed lines after white dots indicate the time till fledgling had a brood remained alive. If death of both chicks occurred on the same day, only one dot is visible on a line. Although no invertebrate data were available after 4 August in 2008 and 8 August in 2009, we assumed these values would be close to zero due to prior trends.

Overall, 61% (*n* = 41) of initial broods had a least one radio-tagged chick survive to 15 days of age (53% in 2008, 68% in 2009), compared to only 33% (*n* = 18) of early replacement broods (31% in 2008, 40% in 2009) and 14% (*n* = 7) of late replacement broods (0% in 2008, 100% in 2009). Chicks died between 1 and 14 days of age and throughout the chick-rearing period (Fig. 2). Both radio-tagged chicks survived to 15 days of age in 29 broods. One radio-tagged chick survived to 15 days of age in 3 broods. Both chicks died in 33 broods, 21 of which had both chicks die during the same 2-day sampling period, while the remaining broods had 1 chick live for at least one additional day (mean time between deaths ± SE = 5.3 ± 0.9 days; range: 1.5–10 days). Of the 131 chicks monitored, 70 (53%) were classified as dead, with 17 (13%) dying from exposure, 7 (5%) being depredated by weasels, 1 (< 1%) being eaten by an avian predator, and 4 (3%) with no obvious cause of death. The single confirmed avian predation event and all weasel predations occurred in 2008. Predation events occurred throughout the season, with an equal number of predation mortalities (i.e., 4 out of 4) occurring before and after the 15 July. However, exposure mortalities were most common in late July, with 15 out of 17 exposure mortalities occurring after the 15 July. This was especially true in 2008 when temperatures remained below 3 °C for several days (Fig. 2). An additional 41 chicks (31%) were never found but presumed dead because radios were no longer heard, and adults were not exhibiting behaviors indicative of a brood prior to the expected date of fledging. The inability to find presumably dead chicks did not vary based on a chick’s age, occurring relatively consistently from 1 to 14 days after hatch.

### Environmental variables

Invertebrate biomass varied between 0.3 and 25 mg/trap/day in 2008 and 2–37 mg/trap/day in 2009 during the 27 June–4 August time period. The most common invertebrate taxa sampled were from the orders Diptera (true flies, 59% of total individuals, 78% of total biomass) and Araneae (spiders, 34% of total individuals, 12% of total biomass). The remaining four taxa (orders Coleoptera, Hemiptera, Hymenoptera, and Lepidoptera) and their larvae each contributed < 5.0% of the total individuals and < 7% of the total biomass. Invertebrate biomass peaked in early July in both years, after which it generally declined, resulting in low food availability (i.e., < 10 mg/trap/day) for late-hatching and replacement broods (Fig. 2). In contrast, chicks from initial nests generally had greater food availability, especially within the first 5–10 days after hatch. Average daily temperatures were slightly warmer in 2009 (mean ± SE: 6.4 ± 0.6 °C) relative to 2008 (4.4 ± 0.4 °C), but both years were quite variable throughout the brood-rearing period (range: 2008 = 0.3–9.7 °C; 2009 = -0.6–13.9 °C; Fig. 2).

### Chick survival

**Figure 3:**
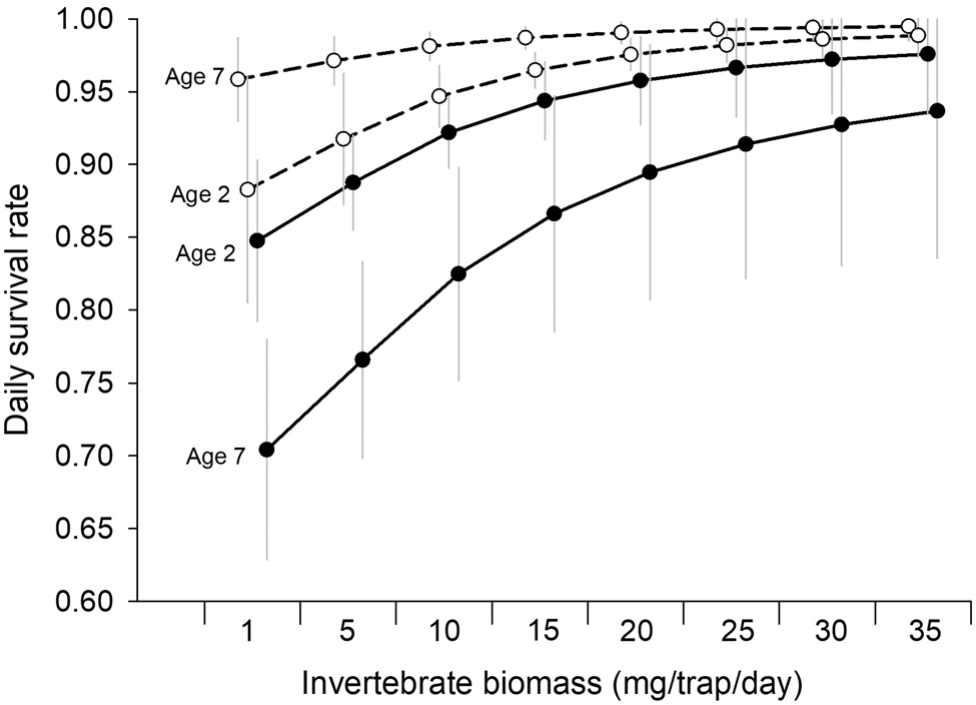
Estimated daily survival rates (± SD) of Dunlin chicks near Utqiaġvik, Alaska, 2008–2009 as a function of invertebrate biomass for chick ages (2 and 7 days) and average hatch date for initial (3 July, dashed lines and open circles) and replacement (15 July, solid lines and closed circles) nests. Daily survival rates and associated SDs were estimated using 10,000 simulations of the beta estimates assuming a multivariate normal distribution and the variance–covariance matrix from the top-ranked model (HD*A^2^ + I; where HD = hatch date, A = chick age, and I = daily invertebrate biomass; Table 1). Symbol locations represent the same invertebrate abundance at each combination of hatch date and age, but symbols are offset so SDs are visible.

**Figure 4:**
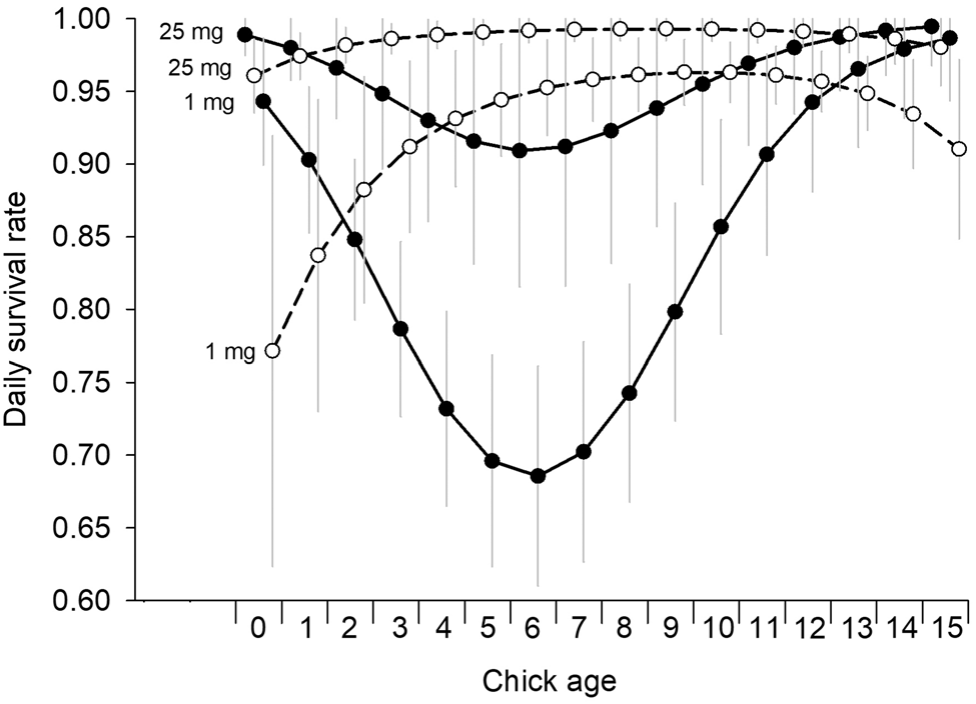
Estimated daily survival rates (± SD) of Dunlin chicks near Utqiaġvik, Alaska, 2008–2009 as a function of chick age for average hatch date for initial (3 July, dashed lines and open circles) and replacement (15 July, solid lines and closed circles) nests, and invertebrate biomass (1 and 25 mg/trap/day). Daily survival rates and associated SDs were estimated using 10,000 simulations of the beta estimates assuming a multivariate normal distribution and the variance–covariance matrix from the top-ranked model (HD*A^2^ + I; where HD = hatch date, A = chick age, and I = daily invertebrate biomass; Table 1). Symbol locations represent the same chick age for each combination of hatch date and invertebrate abundance, but symbols are offset so SDs are visible.

Two models explaining DSR of chicks were considered plausible (ΔAIC_*c*_ < 2; Table 1). However, these models only differed by the inclusion of invertebrate abundance in the top-ranked model, for which the 85% confidence interval did not overlap zero. Therefore, we only present results from the top-ranked model: mean (SE): DSR = − 0.843 (1.548) + 0.244 HD (0.090) + 1.309 A (0.385) − 0.077 A^2^ (0.023) − 0.116 HD*A (0.029) + 0.008 HD*A^2^ (0.002) + 0.082 I (0.049); where HD = hatch date, A = chick age, and I = daily invertebrate biomass. Based on this model, DSR increased as invertebrate biomass increased for chicks at all ages and for chicks hatching earlier in the season (Fig. 3). Chick age exhibited a quadratic relationship with DSR and an interactive effect with hatch date. For illustration, we present results using the average hatch date of initial (i.e., 3 July) and replacement nests (i.e., 15 July). For early-hatching chicks (e.g., 3 July), DSR increased as chicks became older with a slight decline as chicks approached fledging (Fig. 4). In contrast, the DSR of late-hatching chicks (e.g., 15 July) was high in chicks < 1 week old, decreased around 1 week of age, and then increased as they got older (Fig. 4).

**Table 1:** The five most-supported models plus the intercept-only model from a set of 105 candidate models estimating daily survival rates of Dunlin chicks near Utqiaġvik, Alaska, 2008–2009.

Model^a^	K^b^	AIC_*c*_	ΔAIC_*c*_^c^	*w*_*i*_^d^
HD*A^2^ + I	7	196.39	0.00	0.47
HD*A^2^	6	197.62	1.23	0.26
HD*A^2^ + T	7	198.78	2.39	0.14
HD*A^2^ + Y	7	199.65	3.26	0.09
HD^2^*A + I	7	204.86	8.47	0.01
Intercept	1	230.98	34.59	0.00

^a^Variables in the models are hatch date as a linear (HD) or quadratic term (HD^2^), chick age as a linear (A) or quadratic term (A^2^), daily invertebrate biomass (I), daily average air temperature (T), and year (Y). When a quadratic effect was included in a model, we also included the linear effect.

^b^No. of parameters.

^c^Difference between models Akaike’s Information Criterion corrected for small sample size and the lowest AIC_*c*_ value.

^d^AIC_*c*_ relative weight attributed to model.

**Figure 5:**
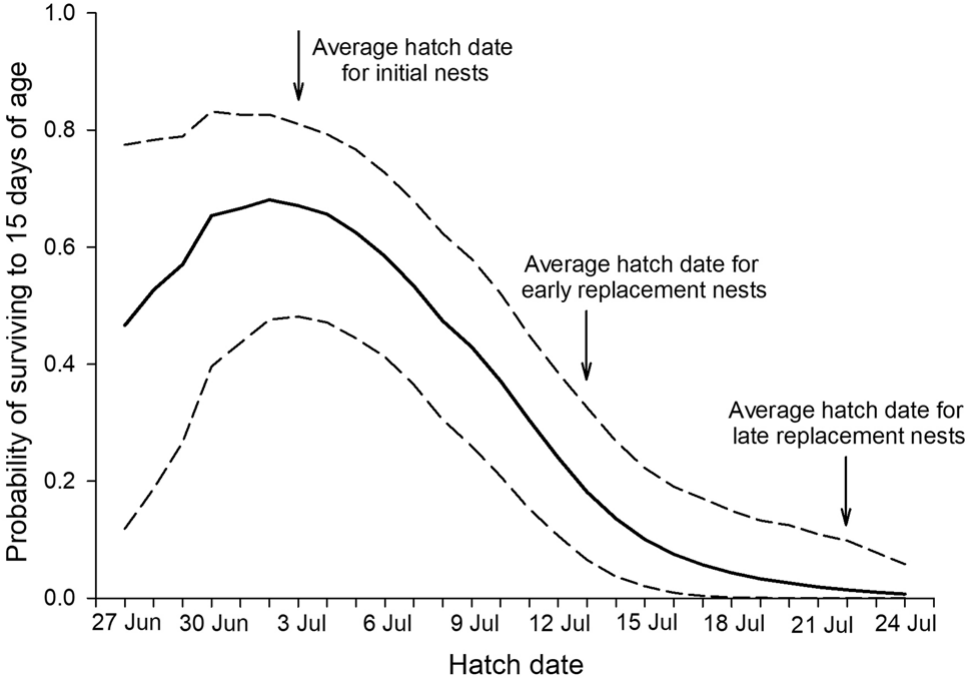
Estimated probability of a Dunlin chick surviving to 15 days of age near Utqiaġvik, Alaska, 2008–2009 as a function of hatch date (solid line; 95% CI shown as dashed lines). Survival probabilities and associated CIs were estimated using average invertebrate biomass estimates based on date and 10,000 simulations of the beta estimates assuming a multivariate normal distribution and the variance–covariance matrix from the top-ranked model (HD*A^2^ + I; where HD = hatch date, A = chick age, and I = daily invertebrate biomass; Table 1). Although no invertebrate data were available after 4 August in 2008 and 8 August in 2009, we assumed these values would be close to zero due to prior trends.

The probability a chick survived to 15 days of age (Ŝ_*i*,15_) was greatest for chicks hatching in late June/early July, declining throughout the rest of the season (Fig. 5). The probability a chick survived to 15 days of age was substantially greater for initial (Ŝ_*i*_ = 0.67; 95% CI = 0.48–0.81) than replacement nests (Ŝ_*i*_ = 0.10; 95% CI = 0.02–0.22; Fig. 5), based on average hatch dates of each group. Note, that we present results using an averaged insect biomass for each day of the season for both years combined (Fig. 5).

## Discussion

Similar to previous studies in subarctic and temperate regions^10,26,31^, our results from an Arctic-breeding site indicate that survival from hatching to fledging was greater for chicks from initial compared to replacement nests. Regardless of nest type, however, our results indicate that an individual can increase their probability of successfully fledging young by nesting early in the season. This may be due to greater food available to chicks hatching early, as late chicks often hatched after invertebrate biomass peaked (Fig. 2). Thus, diminished growth rates of chicks developing during periods of low food availability^6,7,10,21,22,43,44,49^ likely results in greater chick mortality; these results have a direct implication under future climate change scenarios in which greater phenological mismatch is predicted to occur (see below). In addition to benefiting from greater reproductive success, it has been previously suggested that adult birds may also experience strong selection to complete breeding as soon as possible to allow for ample time to rebuild reserves prior to migration^5,15,50^. Therefore, nesting early may afford chicks greater parental care^22^, as well as allow both adults and their chicks increased time to acquire sufficient reserves prior to southbound migration^15,50,51^. Given the numerous benefits, there appears to be strong selection for shorebirds to nest as early as possible; however, reproductive phenology can also be influenced by other factors that can be highly variable both spatially and temporally. For example, factors such as weather conditions or predation rates^9,10,22,51^ that vary seasonally, but often unpredictably, provide inconsistent selection patterns for timing reproduction, often making synchronization of reproduction with ideal conditions difficult.

Daily survival rates were consistently high across all ages for early hatching chicks, except in very young chicks when invertebrate availability was low (Fig. 4). Reduced survival among young chicks has been reported widely, and is presumed to be due to chicks being unable to thermoregulate, and their need to learn to forage efficiently and avoid predators^22,48,52–58^. In contrast, we found young chicks from late broods to have consistently high survival rates, even when food was scarce. The survival of these chicks, however, plummeted as they reached 1–2 weeks of age, but then increased again as they got older (Fig. 4). While we can only speculate why these age-specific survival rates differed between early and late broods, differences may have been due to different environmental conditions (e.g., weather, predation rates, or invertebrate availability) experienced by developing chicks hatching at different times in the season. We also cannot discount the possibility that environmental differences (e.g., the composition and abundance of predators) in the experimental and unmanipulated areas were responsible for some of these differences in age-specific survival rates. Finally, it should be noted that survival differences, especially in the outer extremes of age (i.e., very young and very old chicks; Fig. 4) may have been due to small sample sizes (e.g., early hatching chicks rarely experienced low invertebrate availability while late hatching chicks rarely experienced high invertebrate availability). Despite the differences in age-related patterns in chicks from initial and replacement clutches, it is clear that late hatching chicks survived much worse overall (Fig. 5).

Our estimate of chick survival to fledging for initial nests (67%) was similar to, but generally greater, than those found in studies of other Dunlin subspecies (64% in Finland^59^, 31–60% in northern Sweden^60^, 33.5% in southern Sweden^61^), as well as other small Arctic shorebird species (40–60% in Ringed Plover *Charadrius hiaticula*^62^, 26% in Buff-breasted Sandpiper *Calidris subruficollis*^25^, 43% in Curlew Sandpiper *C. ferruginea*^46^, 73% in Western Sandpiper *C. marui*^22^). However, the removal of arctic fox, an apex predator^63–65^, from our study area suggests our estimates may be artificially high. Other studies have found mixed^66^ or strong positive effects^67^ of predator removal on shorebird chick survival, although none of these studies were conducted on Arctic-breeding shorebirds. The effect of fox removal at our site was difficult to evaluate as the actual number of foxes present was not determined. Further, predation by avian or other mammal (e.g., weasel) species and the abundance of alternative prey (e.g., lemmings) may have masked any benefits from low fox numbers. Our two-year study provides insufficient data to tease apart the relative importance of these factors in regulating chick survival. Clearly, more work is needed to determine the relative impact of predation (by foxes as well as other species) on shorebird chick survival.

The information provided in this study provides valuable demographic information that will help assess the role chick survival may play in limiting population size, even if our estimates of chick survival may be inflated from predator removal. To improve our understanding of how vital rates impact population trends in this species and other shorebird species, additional information on other vital rates (e.g., probability of nesting and hatching, first-year survival, adult survival partitioned by season) and how they vary with site and annual conditions is needed^34,68^. By improving our knowledge and accuracy of shorebird vital rates, we can improve the accuracy of demographic models, as well as determine the cumulative impacts of climate change on shorebird populations (see below). Ultimately, this information will help us better direct where and when conservation actions should be conducted.

Rapid warming at northern latitudes has led to earlier, warmer, longer, and more seasonally-variable summers^33,68^. Such climate change effects may have several positive effects on shorebirds and their young. First, warmer and longer summers might be expected to extend the availability of invertebrate prey. However, entomological studies investigating climate change effects on invertebrate timing and availability suggest otherwise^16,69^. This is because the emergence of most invertebrates begins with the onset of snowmelt and is ultimately determined by the accumulation of temperature degree-days above freezing^70,71^; once adequate temperatures are reached, invertebrates emerge in large pulses that are present for only 1–2 weeks due to their finite life span. Even when invertebrate taxa are combined, invertebrates are only available for about 1–2 months of the 4-month Arctic summer^16,72^. As warmer temperatures occur earlier, the period of prey availability is expected to also shift earlier, but the window of availability will likely remain similar or may even reduce if cumulative temperature degree-days for multiple taxa are reached faster. Furthermore, warmer temperatures may affect the invertebrates themselves, leading to the development of smaller individuals that develop faster but are less fecund^69,73^. Smaller and fewer invertebrates would mean less food available to shorebirds. Second, warmer and longer summers may decrease the energy needed by chicks to thermoregulate^21^, as well as the amount of time adults need to spend brooding. This could increase the time available for chicks to forage^44,74,75^. However, our results and others^6,28^ suggest that temperature is less important to shorebird growth and survival than invertebrate availability. Thus, any positive effects warmer temperatures may provide in physiological relief are likely to be negated by increased phenological mismatch between timing of shorebird hatch and invertebrate availability. Lastly, climate change may benefit shorebirds by lengthening the nesting season. This would allow adults to lay nests over a longer period of time^68^, as well as replace more nests that fail^32^ (but see Cosgrove et al.^76^). However, given the strong relationship between chick survival and invertebrate availability, a longer breeding season is likely to be more detrimental than beneficial due to the increased phenological mismatch with their invertebrate prey (as discussed above). Indeed, a longer, warmer summer may actually result in a shorter period of time in which nesting can be productive. Collectively, these results indicate that any positive effects from warmer, longer summers are unlikely to provide any substantial reproductive benefits to shorebird populations living in the Arctic.

## Methods

### Study species

The *arcticola* Dunlin breeds in northern Alaska (and rarely in northwestern Canada) and winters in Asia, along the coasts of China, Japan, Taiwan, North Korea, and South Korea^77,78^. This subspecies is thought to be declining^34,79^, with recent evidence suggesting the loss of non-breeding habitat^39–42^ is causing low annual adult survival^38^. As a result, this subspecies is of conservation concern in Alaska^80,81^ and throughout its nonbreeding grounds in the East Asia-Australasian Flyway^82^. Dunlin are a monogamous, ground-nesting shorebird that normally lay a 4-egg clutch^83^. While both parents incubate, females typically abandon a few days after hatch, leaving the male to rear the brood. The young are precocial, leaving the nest within a few hours after the last egg hatches. The adults aid in chick thermoregulation, predator detection and evasion, and lead chicks to good foraging habitat, although chicks forage independently.

### Study area

In 2008 and 2009, we monitored chick survival near Utqiaġvik, Alaska (71° 18″ N, 156° 45″ W; Fig. 1). Initial (unmanipulated) nests were predominately located in or near six 36-ha plots in a study area situated to the southeast of Utqiaġvik (hereafter termed unmanipulated area)^84^. The authors have monitored nests and banded adult birds in the study area since 2003 to explore potential limiting factors affecting nest and adult survival and by association shorebird population growth^34,38,84,85^. Nests used in our experimental clutch replacement study, as well as a few initial nests, were located ~ 5–10 km west of these plots (hereafter termed experimental area) to minimize human disturbance to our long-term study. Seasonal phenology and habitat within both study areas were similar. Vegetative structure included a mosaic of low, wet marshes and higher, well-drained uplands (see references in Villarreal et al.^86^).

Potential predators of chicks in Utqiaġvik included Pomarine (*Stercorarius pomarinus*), Parasitic (*S. parasiticus*), and Long-tailed (*S. longicaudus*) Jaegers; Glaucous Gulls (*Larus hyperboreus*); Snowy Owls (*Bubo scandiacus*); Peregrine Falcons (*Falco peregrinus*); Common Raven (*Corvus corax*); least (*Mustela nivalis*) and short-tailed (*M. erminea*) weasels; and arctic foxes (*Vulpes lagopus*). Fox removal occurred throughout the study area in both years to increase the productivity of threatened Steller’s Eiders (*Polysticta stelleri*), although trapping success was lower in 2009 due to a change in fox trapping methods. A summary of how avian predators, lemmings, snow cover, and fox removal varied between the two years can be seen in Figure 6 of Saalfeld and Lanctot^84^. Briefly, the mean number of avian predators observed per plot on point counts, as well as the mean number of lemmings observed per researcher per day were greater in 2008 (avian predators = 9.1 ± 2.4; lemmings = 21.3 ± 3.1) compared to 2009 (avian predators = 3.3 ± 0.8; lemmings = 0.03 ± 0.01)^84^. As we only had two years of data, we were unable to tease apart the relative importance of these factors in regulating chick survival.

### Field methods

Between early June and mid-July we located Dunlin nests by following adults back to nests or by flushing adults as we walked or rope-dragged the study area (see Saalfeld and Lanctot^84^ for more details on methodology). We revisited nests found during laying to determine final clutch size and the start of incubation. We determined the start of incubation by floating eggs for nests found after clutch completion^87^. We predicted hatch date by adding 21 days (incubation period for this species^24^) to the estimated incubation start date (day 0 = date last egg laid). We checked nests every four days during the first 17 days of incubation, and daily near the estimated hatch date to ensure capture of chicks.

We captured adults on nests using a bow-net^88^ and marked them with a single U.S. Geological Survey (USGS) metal leg band, unique combinations of color bands, and a single leg flag. We used morphological measurements to sex adults in the field (females are generally larger than males^89^), and later verified sex assignment with genetic analysis. We attached radio transmitters (Model A2455, 1.2 g, Advanced Telemetry Systems, Isanti, Minnesota; or Model BD-2, 1.4 g, Holohil Systems Ltd., Ontario, Canada) to adults by clipping feathers ~ 1 cm above the uropygial gland and then gluing the radio to the skin with Loctite 454 glue^90^. Radio transmitters were placed on both males and females in the experimental area to determine whether mates remained together to nest again after experimental clutch removal^32^ (see below). We only radio tagged males in the unmanipulated area because males typically care for the young longer, and we were only interested in determining chick survival from these nests.

We removed eggs from initial clutches in the experimental area after both adults were radio tagged during early (3–8 d; *n* = 60) or late (12–16 d; *n* = 29) incubation. Experimental nests were assigned to the early or late removal treatment using a systematic random design to minimize any potential differences in mean initiation dates between groups^32^. No clutches were removed from the unmanipulated area. Although we cannot be certain the first nest found for a pair was their initial nest, it seems likely given these nests were generally found at the beginning of the breeding season, and the large number of banded adults within the unmanipulated area helped us monitor nesting activities. We followed radio-tagged adults in the experimental area after clutch removal to locate respective replacement nests (*n* = 48 for early replacement nests; *n* = 15 for late replacement nests), which we then monitored as described above until chicks hatched.

We caught chicks by hand at or near the nest within 24 h of hatch. We marked chicks with a single USGS metal leg band covered with a single thin piece of colored tape for individual identification within a brood, as previous studies have shown color bands to have little to no effect on chick survival or growth^91^. We attached radio transmitters (Model A2414, 0.3 g, Advanced Telemetry Systems, Isanti, Minnesota; or Model LB-2N, 0.35 g, Holohil Systems Ltd., Ontario, Canada) to two randomly selected chicks per brood using the same technique as for adults^90^. We glued surrounding down feathers over the top of the transmitter to camouflage it and increase tag retention^92^. Transmitters weighed 4.3% or 5.0% (depending on model) of the body mass of a typical 7.0 g chick at hatch. We do not believe transmitter attachment impacted chick survival, given prior studies that indicate impacts are minimal when transmitters are attached with glue and tag weights are minimized^93,94^. We kept chicks warm during handling by placing them in a small cooler heated with chemical packs.

We located chicks every other day until they were found dead or were 15 days of age (hatch day = age 0, so 16 days total, or age of first flight). We located chicks by locating the adult(s) presumably attending the brood, as adult radios emitted a stronger signal and could be heard from a farther distance than chick radio signals. On the rare occasions when the attending parent did not have a radio (e.g., failure to tag the male or when males deserted), we relied solely on chick radio signals to locate them. We retreated after visually locating the radio-tagged adult until it stopped alarm calling and resumed calling to its chicks (similar to ‘gather call’ in Johnson et al.^95^). We then conducted a 5-min observation of the brood to determine the number and identity of chicks, and to confirm the presence of one or both adults. We also recorded whether or not an adult exhibited behaviors indicative of a brood at any time during the visit. Behaviors indicative of a brood included adults sounding alarm calls or performing a “rodent run” distraction display^96^ when an observer approached the brood, then initiating calling with chicks when the perception of “danger” abated. In contrast, behaviors not indicative of a brood included foraging rapidly, preening, roosting, and allowing observers to approach without alarm calling. If a chick was not observed visually, but the radio signal was in the direction of the parent, we considered the chick to be alive and did not approach it. If the radio signal was not in the direction of the parent, we tried to locate the chick to confirm it was alive. If a chick radio signal was not heard, we listened for it in the surrounding area and elsewhere for the remainder of the season to maximize the possibility of relocating a chick that was alive, but separated from its brood, or that had died and whose radio was still transmitting.

We classified chicks as alive if visually observed, if a radio signal was detected near the adult, or if the radio signal was not heard but the attending adult demonstrated behaviors indicative of a brood (we assumed the chick radio fell off or malfunctioned). We classified chicks as dead if a carcass was found, if the radio signal was missing and the attending adult did not exhibit behaviors indicative of a brood for two consecutive visits, or if the radio signals for both the adult and chick went missing prior to 15 days of age and were not heard for the remainder of the season. We considered chicks found dead but intact on the surface of the tundra with no apparent flesh wounds to have died of exposure, chicks found in burrows with gashes or bite marks to have been depredated by weasels, and any chick whose radio was found in an avian pellet to be depredated by an avian predator. Unfortunately, it was not possible to determine whether a chick died from exposure before being depredated. For chicks found dead, we assumed date of death was the median date between the last date seen alive and date found dead.

We used pitfall traps to estimate available invertebrate biomass during the chick-rearing period following Tulp and Schekkerman^15^. We established one transect in each of the experimental and unmanipulated study areas and placed 10 traps along each transect spaced 20 m apart, five in mesic habitat and five in xeric habitat. We constructed traps out of 473 ml (16 oz) clear plastic drinking cups that were cut to ~ 9 cm in height, resulting in an 8 cm diameter opening. We filled each trap with ~ 2 cm of water and a few drops of laundry detergent, which reduced surface tension and prevented invertebrates from escaping. We then placed each trap into the tundra so that the rim was flush with the ground. We collected invertebrates from the traps every other day from 23 June to 4 August in 2008 and 21 June–8 August in 2009. We measured invertebrate lengths to the nearest 0.5 mm for individuals < 5.0 mm and to the nearest 1.0 mm for individuals > 5.0 mm^15^. We identified adult and larval invertebrates to order, and converted lengths to a dry mass weight (mg) using regression models developed for Araneae, Coleoptera, Diptera, Hemiptera, Hymenoptera, and Lepidoptera by Rogers et al.^97^. We then summed individual biomasses daily for all invertebrate taxa across all 20 traps, and then divided this by 20 (total number of traps) and 2 (to control for the 2-day sampling period) to estimate total invertebrate biomass per trap day. This approach assumes equal invertebrate biomass on the collection and non-collection days. We calculated average daily air temperatures (i.e., average of the minimum and maximum temperature for a given day) using values from the Wiley Post-Will Rogers Memorial Airport in Utqiaġvik, which was located between 2 and 10.5 km from the experimental and unmanipulated study areas (National Climate Data Center; www.ncdc.noaa.gov).

All procedures were performed in accordance with relevant guidelines and regulations, approved by the Institutional Animal Care and Use Committee at the University of Alaska, Fairbanks, USA (IACUC Assurance 08-12), the Alaska Department of Fish and Game, Juneau, USA (Scientific Permit 08-122 [2008] and 09-011 [2009]), and the U.S. Fish and Wildlife Service (Permit MB088686-0), as well as in adherence to the ARRIVE guidelines.

### Data analysis

We used a generalized linear model with logit link for nest survival implemented in program MARK (Version 8.2)^98^ to estimate daily survival rates (DSR) of chicks^99^:

$$DSR_{i} = \frac{{\exp \left( {\beta_{0} + \mathop \sum \nolimits_{j} \beta_{j} x_{ji} } \right)}}{{1 + \exp \left( {\beta_{0} + \mathop \sum \nolimits_{j} \beta_{j} x_{ji} } \right)}}$$

where $\beta_{0:j}$ are coefficients to be estimated from the data and $x_{ji}$ are measurements of covariate *j* on day *i*. Nest survival models are a type of known fate model (i.e., detection probability is not estimated), which allows staggered observations and an unknown day of death^100^. Nest survival models assume that: (1) chicks are correctly aged on the first visit, (2) fates are known with certainty, (3) chicks are independent, (4) brood checks do not influence survival, and (5) survival among chicks is homogeneous^100^. We are confident that the first assumption was met because we checked nests daily near the expected hatch date, and chicks were banded in the nest bowl or close by. Certainty of fates may not have been met in all cases, but we attempted to minimize error in assigning fates by basing fate on a combination of indicators: presence of a carcass, adult behavior, and presence/absence of a radio signal. Although this may have failed in some instances (e.g., where chicks were adopted by another brood or abandoned by their parents causing parents not to exhibit behaviors indicative of a brood, or if parents continued to exhibit behaviors indicative of a brood after chicks had died); these situations were likely rare (see Hill^101^ for more discussion on this topic). Additionally, we may have incorrectly classified the fate of a chick in instances where we relied only on adult behavior (i.e., adult exhibited behaviors indicative of a brood but no chick radio signal was heard). In these instances, at least one chick within the brood was likely alive, although the monitored chick may have died. As the majority of chicks within a brood died on the same day (see “Results” section), we believe this method accurately classified fate in most instances. However, to ensure independence in the sample and reduce the likelihood of under-inflated sampling variances, we randomly selected one of the two monitored chicks from each brood to include in the analysis. We chose this approach rather than including brood as a random effect in our analysis, as the majority of chicks within a brood had identical encounter histories. We assumed brood checks did not influence survival because most brood checks were conducted at a distance that appeared not to cause disturbance (e.g., after contact calling resumed, chicks performed normal behaviors). To account for non-homogeneity in chick survival, we included covariates expected to cause differences in survival among chicks such as invertebrate biomass, temperature, year, age, and hatch date.

We used an information-theoretic approach^102^ for model development and selection. We developed a set of models using five variables observed to influence chick survival in prior studies: daily invertebrate biomass (I), daily average air temperature (T), year (Y), chick age as a linear (A) or quadratic (A^2^) function, and hatch date as a linear (HD) or quadratic (HD^2^) function. Although we predicted chick survival to increase as chicks aged and earlier in the season, we included quadratic effects for age and hatch date to test for any non-linear relationships in which survival declines for the earliest- or latest-laid nests, or for the youngest and oldest chicks. Note that when a quadratic effect was included in a model, we also always included the linear effect. We used hatch date rather than a categorical variable of the three nest categories (i.e., initial, early replacement, or late replacement) to maximize the use of information in the data. Year was included to account for any potential annual variation.

Our model set included all combinations of single, two, and three variable models with additive and/or interactive effects, with the following exceptions. We excluded models with an interactive effect of year and invertebrate biomass as we assumed invertebrate biomass would have the same effect on chicks regardless of year. Second, we excluded models with interactive effects of daily average temperature with all variables other than chick age as we assumed young chicks were disproportionately affected by cold temperatures, but temperature was likely to affect the remaining variables similarly. We did not consider models with > 3 factors, with > 3-way interactions, or that had quadratic functions for both age and hatch date in an effort to avoid overfitting. Invertebrate biomass and daily average air temperature were not highly correlated in our data set (*r* = 0.063; VIF = 1.00) and were therefore allowed to occur in the same model. The final model set included 105 models. We considered the model with the lowest AIC_*c*_ (Akaike’s Information Criterion corrected for sample size) value to be the best-fitting and models with a ΔAIC_*c*_ < 2 to be plausible^102^. Variables within models were considered important if 85% confidence intervals for the beta estimates did not overlap zero^103^.

To estimate DSR for nests in relation to predictor variables, we conducted 10,000 multivariate normal distribution draws using the estimated beta means and variance–covariance matrix from the top-ranked model. This approach was similarly used to estimate survival from hatch to fledging (i.e., age 15 days) throughout the season:

$${\hat{\text{S}}}_{i,15} = {\text{DSR}}_{i,0} {\text{DSR}}_{i,1} {\text{DSR}}_{{{\text{i}},2}} \ldots {\text{DSR}}_{i,15} ,$$

where *i* is hatch day and subscripts 0–15 are chick ages.

## Data Availability

Long-term shorebird data from Utqiaġvik, Alaska from 2003 to 2018 are available from the National Science Foundation’s Arctic Data Center at 10.18739/A23R0PT35. For specific data pertaining to this paper please contact the authors.
